# Reduced circulating growth hormone and insulin-like growth factor-1 and delayed growth of premature rats are aggravated by longer daily duration of chronic intermittent hypoxia exposure

**DOI:** 10.3389/fped.2022.1008282

**Published:** 2023-01-09

**Authors:** Chi Zhang, Xiaowan Du, Jingjing Li, Junbo Zhang, Guoping Yin

**Affiliations:** ^1^ Department of Otolaryngology, Head and Neck Surgery, Peking University First Hospital, Beijing, China; ^2^ Department of Otolaryngology, Head and Neck Surgery, Beijing Tsinghua Changgung Hospital, School of Clinical Medicine, Tsinghua University, Beijing, China

**Keywords:** insulin-like growth factor-1, chronic intermittent hypoxia, growth delay, rat, growth hormone

## Abstract

**Objective:**

This study mainly aimed to investigate the effect of daily duration of chronic intermittent hypoxia (CIH) exposure on circulating growth hormone (GH)/insulin-like growth factor-1 (IGF-1) concentrations and body weight changes of premature rats.

**Methods:**

40 healthy male SD rats aged six weeks were enrolled in this study. These rats were randomly divided into four groups (*n* = 10 per group), including normal control (NC) group (normal oxygen exposure every day), CIH-1 group (daily CIH exposure for 2 h), CIH-2 group (daily CIH exposure for 4 h), and CIH-3 group (daily CIH exposure for 8 h). The serum GH/IGF-1 concentrations and body weights in all rats were determined after 30 days of normal oxygen or CIH exposure.

**Results:**

No significant difference was found with respect to the baseline body weight among the four groups of rats. After establishments of animal models with a duration of 30 days, significant differences were found respect to body weight, body weight changes, and serum GH/IGF-1 concentrations among the four groups of rats with a same trend (all *P* < 0.05): the highest values were all in NC group rats, followed CIH-1 group, CIH-2 group, and CIH-3 group rats. Among all the rats, the body weight changes correlated significantly with both serum GH and IGF-1 concentrations (both *P* < 0.05).

**Conclusion:**

CIH decreases circulating GH/IGF-1 concentrations and causes growth delay in premature rats. Such effects could be aggravated by increased daily duration of CIH exposures.

## Introduction

Obstructive sleep apnea (OSA) is characterized by recurrent episodes of partial or complete collapse of upper airway during sleep. The physiological consequences of this disease include sleep fragmentation, intermittent hypoxia, increased sympathetic activation, and nocturnal arousal (1). In children, the prevalence of OSA could reach to 3%–10% (2), and this disease is associated with several comorbidities, including decreased quality of life, worsened mental health, increased cardiovascular problems, and poor growth and development (3–6).

Growth hormone (GH) is a peptide hormone synthesized and secreted by the pituitary gland and its primary function is to promote somatic growth mostly through inducing synthesis of insulin-like growth factor-1 (IGF-1) (7), which are vital for skeletal growth in children (8). Reduced circulating GH/IGF-1 concentrations had been reported to be associated with growth delay in children with OSA (9, 10). In addition to sleep fragmentation, OSA related intermittent hypoxemia is also believed to be an important contributor (11, 12). However, the effect of different daily durations of hypoxemia on serum GH/IGF-1 concentrations is unknown. This is important for therapeutic indication, as hypoxemia always present during part of the whole night sleep in the vast majority of children with OSA.

In current study, immature Sprague Dawley (SD) rats were randomly grouped and subjected to different daily durations of chronic intermittent hypoxia (CIH) environment to simulate OSA associated hypoxemia. The serum GH and IGF-1 concentrations, and the body weight changes were determined and compared between different groups of rats. In particular, it was expected to investigate the effect of different daily duration of CIH exposure on serum GH and IGF-1 concentrations and delay growth of premature rats.

## Materials and methods

### Experimental animals

40 healthy male SD rats aged six weeks (obtained from Beijing Vital River Laboratory Animal Technology Co., Ltd.) were used in this study. All the rats were allowed free access to food and water, and raised under the conditions of 12 h light/dark cycle, 22 ± 2°C and 40% humidity. The protocol was approved by the Animal Ethics Committee of Peking University First Hospital. The experiments were performed in accordance with the relevant provisions of Regulations of the People's Republic of China on the Administration of Laboratory Animals.

### Establishments of CIH models of rats

The exposures of CIH in this study were achieved in a hypoxia chamber, which could be adjusted. 40 SD rats were randomly divided into 4 groups (*n* = 10 per group), including one normal control (NC) group (normal oxygen exposure every day) and three CIH groups. The exposure conditions of the three CIH groups were as follows:

(1) CIH-1 group: The rats were persistently exposed to cycles of 10%–21% O_2_ (10% O_2_ for 1 min and 21% O_2_ for 1 min) in the controlled hypoxia chamber for 2 h per day, 30 days in total;
(2) CIH-2 group: The exposure of CIH and the total duration were both the same with CIH-1 group except the daily exposure duration which was increased to 4 h;
(3) CIH-3 group: The exposure of CIH and the total duration were both the same with CIH-1 and CIH-2 groups except the daily exposure duration which was increased to 8 h.

### Body weight measurement and detection of circulating Gh and IGF-1 concentrations

At day 0 and day 30, the weights of all rats were measured and recorded, respectively. At day 30, all the rats were intraperitoneally anesthetized with 4 ml/kg 10% chloral hydrate (0.4 g/kg), and arterial blood samples were harvested from the right femoral artery, respectively. Serum was separated by centrifugation (Eppendorf, Germany) at 3,000 rpm for 15 min and stored at −80°C until analyzed. The circulating GH and IGF-1 concentrations were evaluated by commercially-available ELISA kits (Boster, China), according to the manufacturer's instructions, respectively. The optical density of each well was determined at 450 nm within 30 min.

### Statistical analysis

SPSS 20.0 statistical software (SPSS, Inc., Chicago, IL, United States) was used for data processing. Continuous variables were presented as mean ± standard deviation. Comparisons between the groups were evaluated by One-way ANOVA test. Correlation analysis between different variables were evaluated by Pearson correlation test. *P* < 0.05 indicated a significant difference.

## Results

After establishments of animal models with a duration of 30 days, only one rat in NC group died before blood sample collection. Therefore, the data of the remaining 39 rats were collected for further analysis, including 9 cases in NC group and 10 cases in each CIH group.

**Figure 1:**
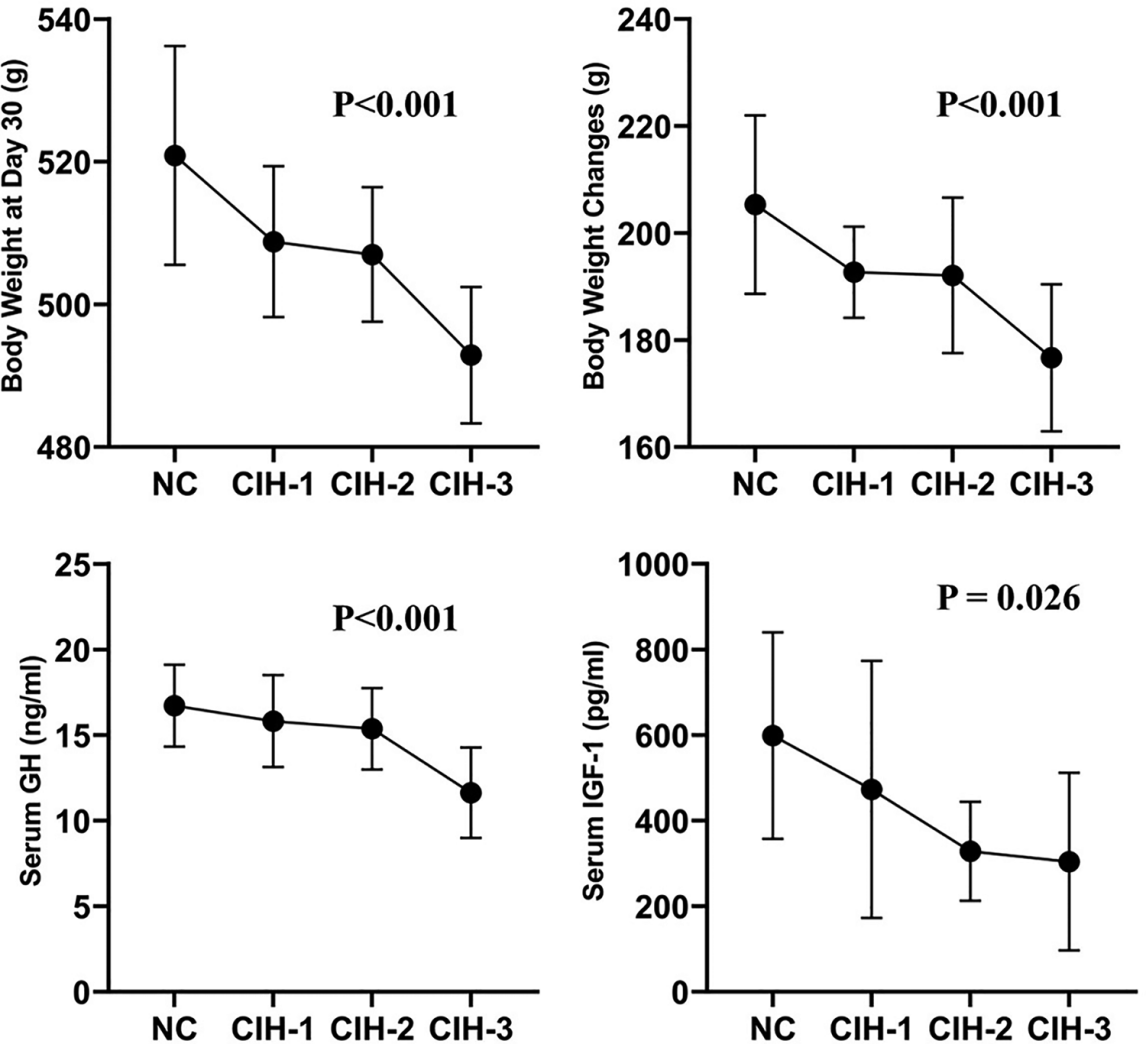
Body weight measured at day 30, body weight changes, and serum GH and IGF-1 concentrations detected at day 30 all differed significantly among the four group of rats in a same trend (all *P* < 0.05): the highest values were all in NC group rats, followed CIH-1 group, CIH-2 group, and CIH-3 group rats.

The body weights measured at day 0 and day 30, the body weight changes, and the serum GH and IGF-1 concentrations detected at day 30 were shown in Table 1: No significant difference was found in baseline body weight among the four groups of rats (*P* = 0.987). However, there were significant differences respect to body weight measured at day 30, body weight changes, and serum GH and IGF-1 concentrations detected at day 30 (all *P* < 0.05). Furthermore, the trend was same: the highest values were all in NC group rats, followed CIH-1 group, CIH-2 group, and CIH-3 group rats (as shown in Figure 1).

**Table 1:** The body weight, body weight changes, and serum GH/IGF-1 concentrations among the four group of rats after 30 days.

	NC (*n* = 9)	CIH-1 (*n* = 10)	CIH-2 (*n* = 10)	CIH-3 (*n* = 10)	*P* value
Body Weight at day 0 (g)	315.6 ± 3.8	316.1 ± 11.1	314.9 ± 12.0	316.2 ± 5.7	0.987
Body Weight at Day 30 (g)	520.9 ± 15.3	508.8 ± 10.6	507.0 ± 9.4	492.9 ± 9.6	<0.001
Weights Changes (g)	205.3 ± 16.7	192.7 ± 8.5	192.1 ± 14.5	176.7 ± 13.7	<0.001
Serum GH (ng/ml)	16.7 ± 2.4	15.8 ± 2.7	15.4 ± 2.4	11.6 ± 2.6	<0.001
Serum IGF-1 (pg/ml)	599.0 ± 241.3	473.1 ± 300.3	328.6 ± 115.4	304.3 ± 207.5	0.026

**Figure 2:**
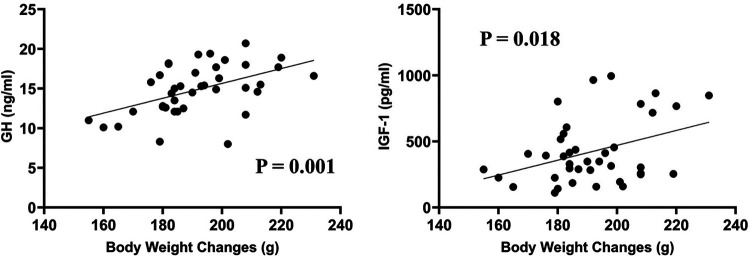
Among all the 39 rats, the body weight changes correlated significantly with both serum GH (correlation coefficient = 0.495, *P* = 0.001) and IGF-1 concentrations (correlation coefficient = 0.377, *P* = 0.018).

Among all the 39 rats, further analyses showed that the body weight changes correlated significantly with both serum GH (*P* = 0.001) and IGF-1 concentrations (*P* = 0.018) (as shown in Figure 2), with correlation coefficients of 0.495 and 0.377, respectively.

## Discussion

GH/IGF-1 axis plays essential roles in regulating multiple physiological processes in humans and other species, including in promoting somatic growth in children (13–17). The present study showed that daily CIH exposure could surely reduce circulating GH/IGF-1 concentrations and cause growth retardation *in vivo,* which supported the findings of former clinical studies (18–21). On the other hand, the significant correlations between circulating GH/IGF-1 concentrations and the body weight changes of the rats confirmed the decisive role of this axis in affecting somatic growth caused by CIH. Therefore, the circulating GH/IGF-1 concentrations could be used as reliable indicators to evaluate the effect of OSA on children's growth and development.

In normal children, the maximal GH secretory usually burst during the period of SWS (17, 22). Thus, individuals with OSA, who demonstrate polysomnographic reduction of SWS, may theoretically display alterations in GH/IGF-1 secretions (22). The current results suggested that circulating GH/IGF-1 concentrations could be directly affected by intermittent hypoxemia, another typical pathophysiological change of OSA. Such effect existed even in a short daily duration of CIH exposure, and became more pronounced with prolonged daily durations of CIH exposures. The pathophysiological basis for this time dependency is unknown. However, the clinical implicating is that, even a short duration of sleep hypoxemia per day (two hours in this study) could be proper indication for intervention respect to maintain a normal growth and development.

In clinical work, an in-laboratory polysomnography is not always prescribed for children patients with potential OSA due to its poor compliance, and a nocturnal pulse oximetry has been commonly used as an alternative test. The current results suggested that the latter may also be valuable for evaluating treatment indication, as it could detect the existence of hypoxemia and its duration.

Besides OSA, there were several factors that may affect GH/IGF-1 levels, such as age, genetics, psychological factors, exercise, obesity, nutritional intake, smoking and alcohol status, and even sleep duration (8, 14, 17, 23–27). Therefore, it is hard to exactly explore the effect of OSA related hypoxemia on these two hormone levels in clinical studies. In the present study, rats which had the same species, gender, age, body weights, and diet without smoking and alcohol were enrolled in the study, and the potential bias induced by other factors was excluded to the greatest extent, indicating that our findings above are convincing.

There are some limitations that need to be addressed. First, the number of rats used was relatively small. However, the potential confounders could be well controlled. Second, besides body weight, there were also some other items that could represent growing development, such as skeletal development, cognitive competence, etc., which were not discussed in current study. Third, the specific mechanism by which CIH affects circulating GH/IGF-1 concentrations was not explored in current study. The last two points are the focus of our future research.

## Conclusions

In summary, our study demonstrated that CIH exposure could decrease circulating GH/IGF-1 concentrations and cause delayed growth in premature rats. The decrease of these two hormones and the delayed growth were in same trends. Moreover, longer daily durations of CIH exposures aggravated the above effects.

## Data Availability

The raw data supporting the conclusions of this article will be made available by the authors, without undue reservation.
